# Real-world effectiveness and safety of dupilumab therapy in children ≤6 years with uncontrolled persistent asthma: a propensity-matched retrospective cohort study

**DOI:** 10.3389/fped.2026.1808733

**Published:** 2026-06-15

**Authors:** Ahlam Mazi, Azizah Aljohany, Michele Fouad, Hanaa Hamadallah, Esraa M. Bukhari, Doaa Bayomi, Samahir Alsulaimani, Nour Gazzaz, Muhannad Sharara, Monica Dobs

**Affiliations:** 1 Department of Pediatrics, Faculty of Medicine, King Abdulaziz University, Jeddah, Saudi Arabia; 2 Department of Pediatrics, King Abdulaziz University Hospital, King Abdulaziz University, Jeddah, Saudi Arabia; 3 Child and Women Health Department, Faculty of Medicine, Taibah University, Medina, Saudi Arabia; 4 Faculty of Medicine, Alexandria University, Alexandria, Egypt; 5 Department of Pediatrics, Faculty of Medicine, King Abdulaziz University, Rabigh, Saudi Arabia; 6 Department of Pediatrics, Medical College of Georgia, Augusta University, Augusta, GA, United States; 7 Department of Pediatrics, Serra Medical Clinic, Los Angeles, CA, United States

**Keywords:** asthma, biological treatment, dupilumab, monoclonal antibodies, preschool

## Abstract

**Background:**

Asthma is common in children. Dupilumab is a biological agent that showed effectiveness in older children with uncontrolled asthma; however, its efficacy and safety are lacking among the very young children (≤6) who were excluded from clinical trials.

**Methods:**

Using TriNetX, children ≤6 years old with persistent asthma who received a concomitant prescription for dupilumab for any indication, in addition to standard asthma treatment, were compared with those on standard treatment alone. Using 1:1 propensity score matching across 35 covariates, outcomes such as acute exacerbations, status asthmaticus, need for oral corticosteroid (OCS), emergency room (ER) visits, anaphylaxis, infections, and inpatient admissions were assessed at 6 and 12 months.

**Results:**

Of 44,952 patients analyzed, 301 patients were matched per group. Asthmatic children with concomitant dupilumab prescriptions were associated with a reduction in acute exacerbations at 6 and 12 months (RR 0.53 and 0.46, respectively; *p* < 0.05), OCS use (RR 0.67 and 0.66; *p* < 0.05), ER visits, and infections (all *p* < 0.05). However, the risk of anaphylaxis was higher in cohort one at 6 and 12 months (RR 1.80 and 1.74; *p* < 0.05). Other outcomes, such as status asthmaticus, eosinophilia, and inpatient admissions, were similar between the cohorts.

**Conclusions:**

This real-world analysis shows dupilumab therapy alongside standard treatment in children ≤6 years with persistent asthma is significantly associated with reduced acute exacerbations, OCS use, and ER visits. These findings support dupilumab's clinical benefit for this vulnerable group, aiding clinical decisions.

## 1 Introduction

### 1.1 Background

Asthma is among the most prevalent chronic respiratory disorders in childhood, affecting nearly one in twelve children in the United States and accounting for an estimated seven million pediatric cases nationwide (1, 2). Among pediatric patients, 5%–10% have moderate-to-severe asthma, which is associated with increased risk for abnormal lung growth and possibly the development of chronic obstructive pulmonary disease (COPD) in the future (1). The pathophysiology of asthma in preschool children differs from that in older age groups, with a higher prevalence of virus-induced wheezing and phenotypes prone to exacerbations. In contrast, asthma in older children more often presents as an impairment-dominant condition linked to allergic sensitization (3–7). In preschoolers, symptoms are typically episodic and triggered by viral infections, driven more by airway structural changes and infection-related mechanisms rather than by type 2 inflammation commonly seen in older children (4–6). Diagnostic evaluation in young children is challenging due to the limited ability to perform objective lung function testing, necessitating a reliance on symptom patterns, risk profiles, and treatment response rather than a definitive mechanistic assessment (3, 6).

The standard of care for pediatric asthma is age-dependent. In children aged 6 years and older, guideline-directed therapy encompasses inhaled corticosteroids (ICS), long-acting *β*₂-agonists (LABA), and leukotriene receptor antagonists (LTRA). However, for children under 6 years — particularly those younger than 4 — current GINA guidelines recommend daily low-dose ICS as the preferred first-line controller therapy, with step-up options prioritizing increased ICS dose or the addition of LTRA (8). LABA use is not routinely recommended in this age group and is generally reserved for exceptional circumstances under specialist supervision, given limited safety and efficacy data in young children (8). Most regulatory approvals for ICS-LABA combinations are restricted to children aged 4 years and older, and prescribing these agents in younger children falls outside licensed indications in the majority of jurisdictions (8). Although these agents provide effective control for many patients, a subset of children remains inadequately managed despite maximal guideline-directed therapy necessitating escalation to higher ICS doses or systemic corticosteroids, which carry significant adverse effects (1, 9, 10). The American Academy of Pediatrics recommends consideration of biologic agents for children with persistent symptoms, while the Global Initiative for Asthma (GINA) extends this recommendation to steps 4 and 5 of the stepwise management framework specifically in children aged six years and older (2, 8). Biologic therapies have transformed the management of uncontrolled asthma, providing targeted treatment options for children—particularly those with type 2 inflammatory phenotypes. These phenotypes are typically characterized by elevated blood eosinophil counts, increased fractional exhaled nitric oxide (FeNO), and evidence of allergic sensitization (1, 2, 11). Dupilumab, a fully human monoclonal antibody, inhibits the shared receptor component for interleukin-4 (IL-4) and interleukin-13 (IL-13), two key cytokines that drive type 2 inflammation. By blocking these pathways, dupilumab has been shown to reduce biomarkers such as FeNO and serum immunoglobulin E (IgE), while improving percent predicted forced expiratory volume in one second (ppFEV₁), decreasing exacerbation frequency, and enhancing health-related quality of life in children aged 6–11 years (1, 2, 9, 10, 12–15). Reported adverse events are generally mild, most commonly injection-site reactions, with infrequent occurrences of symptomatic eosinophilia (1, 2).

Although dupilumab has demonstrated clear benefits in patients with type 2 asthma, its efficacy in preschool children remains uncertain (9, 10). Despite these advances, children under six years have been consistently excluded from all pivotal trials and systematic reviews, leaving a significant gap in our understanding regarding the efficacy, safety, and optimal use of dupilumab in this age group (1, 2, 11, 12). There is an urgent need for real-world evidence to inform clinical decision-making for children ≤6 years of age with uncontrolled asthma.

### 1.2 Objectives

This study aims to quantify the real-world effectiveness and safety of dupilumab therapy in children aged ≤6 years with asthma that remains uncontrolled despite standard therapies, by comparing outcomes between children who received dupilumab alongside standard care and those on standard care alone. The analysis was done using the TriNetX global health research network, which provides a large, diverse, and representative cohort, thereby enhancing the external validity and generalizability of the findings. By leveraging this database, the study addresses limitations of prior clinical trials related to age restrictions and demographic representation, delivering urgently needed evidence to guide the use of dupilumab in young children with asthma. Outcomes included real-world effectiveness and safety, specifically acute exacerbations, emergency room visits, status asthmaticus, and the need for oral corticosteroid use.

## 2 Methods

This study was exempt from institutional review board approval and informed consent requirements, as it constitutes a secondary analysis of existing de-identified data that does not involve intervention or interaction with human subjects, in accordance with Section §164.514(a) of the HIPAA Privacy Rule. The study adhered to the Strengthening the Reporting of Observational Studies in Epidemiology (STROBE) guidelines and the Publication Guidelines specified by the TriNetX platform (16, 17)

### 2.1 Data source and study design

This retrospective cohort study utilized data collected on 25 August 2025 from the TriNetX Global Network (17), a federated real-world data platform that aggregates de-identified electronic medical records — including diagnoses, procedures, medications, laboratory values, and genomic information — from healthcare organizations across the United States, encompassing hospitals, outpatient clinics, and ambulatory care settings. The dataset covers both insured and uninsured patients and, at the time of data extraction, included information on approximately 155 million individuals from 106 healthcare organizations. TriNetX employs natural language processing to enhance data capture from clinical notes. All data are mapped to standardized coding systems such as Current Procedural Terminology (CPT) for procedures, International Classification of Diseases (ICD) for diagnoses, RxNorm for medications, Logical Observation Identifiers Names and Codes (LOINC) for laboratory results, and benraL7V3.0 for visit data. All patient data are deidentified in accordance with Section §164.514 of the Health Insurance Portability and Accountability Act (HIPAA) Privacy Rule, and participating organizations maintain compliance with these standards.

### 2.2 Participants' selection criteria

Children aged ≤6 years with persistent asthma receiving standard baseline therapy, including ICS (beclomethasone, budesonide, ciclesonide, or fluticasone propionate), LABAs (formoterol or salmeterol), and/or LTRA montelukast, were included and stratified into two cohorts. Persistent asthma was defined according to ICD-10-CM diagnostic codes: mild persistent (J45.30–J45.31), moderate persistent (J45.40–J45.41), and severe persistent (J45.50–J45.51), as recorded in the EHR by the treating clinician. TriNetX does not capture the diagnosing clinician's specialty; therefore, diagnoses may have been made by general pediatricians, pediatric allergists, or pulmonologists. We acknowledge that asthma diagnosis in children younger than six years remains inherently challenging, and an overlap with recurrent viral wheezing phenotypes that do not represent true persistent asthma cannot be excluded. Patients receiving dupilumab alongside standard therapy (DUP group) represented cohort one, and those on standard treatment alone (ST-ONLY group) cohort two Dupilumab was not licensed for asthma in children ≤6 years at the time of this analysis. In this age group, its approved indications encompass moderate-to-severe atopic dermatitis and eosinophilic esophagitis (EoE), and prescriptions captured in the DUP cohort may reflect either or both of these indications — or a broader clinical decision to suppress shared type 2 inflammatory pathways across coexisting atopic conditions (18–20), As the driving indication cannot be ascertained at the encounter level in TriNetX, this remains an inherent analytical limitation. Eosinophilic esophagitis was not extracted as a baseline variable and was not incorporated as a covariate in the propensity score matching model; its prevalence in either cohort therefore cannot be reported. That said, atopic dermatitis prevalence was comparable across both matched cohorts (DUP: 76.4% vs. ST-ONLY: 81.1%; SMD 0.114), and roughly one in four dupilumab-treated children carried no such diagnosis, suggesting asthma may have been the primary driver in a meaningful subset. The balance of atopic and non-atopic patients across both arms therefore supports, rather than undermines, the robustness of the observed findings. LABA prescriptions were similarly captured as documented in the EHR and could not be linked to prescribing specialty or clinical indication; compliance with current GINA guidance for children under 6 years could therefore not be confirmed. More broadly, medication data in TriNetX reflect prescription records rather than confirmed dispensing or patient adherence, and a single recorded prescription was sufficient for treatment classification — limiting certainty regarding actual therapeutic exposure. Patients who were receiving any other biologics (Tezepelumab, mepolizumab, omalizumab) or follow-up less than one year were excluded from both cohorts. The inclusion and exclusion criteria, as well as the codes used to identify the cohorts, are outlined in Supplementary Table S1.

### 2.3 Baseline characteristics and propensity score matching

To simulate randomized conditions, a 1-to-1 propensity score matching (PSM) using the nearest-neighbor method with a caliper of 0.1 pooled standardized mean difference (SMD) was performed across 35 baseline covariates, including demographics, diagnoses, medications, and relevant laboratory values. To mitigate selection bias, disease burden comparability was ensured by adjusting for asthma severity (mild, moderate, severe), complications (uncomplicated, with acute exacerbations, status asthmaticus), associated other allergic conditions (atopic dermatitis and allergic rhinitis), and previous prescriptions of OCS (prednisolone and prednisone). Food allergy was not included as a propensity score matching covariate, representing a potential source of residual confounding, particularly with regard to the anaphylaxis outcome. Laboratory parameters were also considered, including eosinophil-based risk indicators (mild: <150 cells/µL, moderate: 150–299 cells/µL, severe: ≥300 cells/µL), as well as IgE levels. Details of the codes used to identify the baseline upon which the PSM was performed are provided in Supplementary Table S2.

### 2.4 Study endpoints

Outcomes included acute asthma exacerbations, need for OCS (prednisolone or prednisone), and emergency room (ER) visits, status asthmaticus, inpatient admissions, infections, eosinophilia, and anaphylaxis. The infections endpoint was a composite of acute upper respiratory infections (ICD-10 J00–J06), bacterial infections of unspecified site (A49), and viral infections of unspecified site (B34). Patients were further stratified into eosinophil-based risk groups: low (<150 cells/µL), intermediate (150–299 cells/µL), and high (≥300 cells/µL). The index event for the DUP cohort was the date of the first recorded dupilumab prescription occurring within one year after a qualifying persistent asthma diagnosis. For the ST-ONLY cohort, the index event was the date of the first recorded qualifying standard-therapy prescription within an equivalent temporal relationship to the asthma diagnosis. All outcomes were then assessed at 6 and 12 months from this index date. Owing to the de-identified nature of the TriNetX dataset, continuous verification of dupilumab exposure throughout the follow-up period was not possible; dropout or treatment discontinuation cannot be excluded (Supplementary Table S3).

### 2.5 Statistical analysis

Outcomes were compared between the two cohorts before and after PSM using the “Compare Outcomes Analytic” function of TriNetX. Continuous variables were reported as mean ± standard deviation (SD), and categorical variables were expressed as frequencies and percentages. Risk ratios (RRs) and absolute risk differences (RDs) with 95% confidence intervals (CIs) were calculated for all dichotomous outcome variables. Statistical significance was defined as *p* < 0.05**.**

## 3 Result

### 3.1 Study population

**Figure 1:**
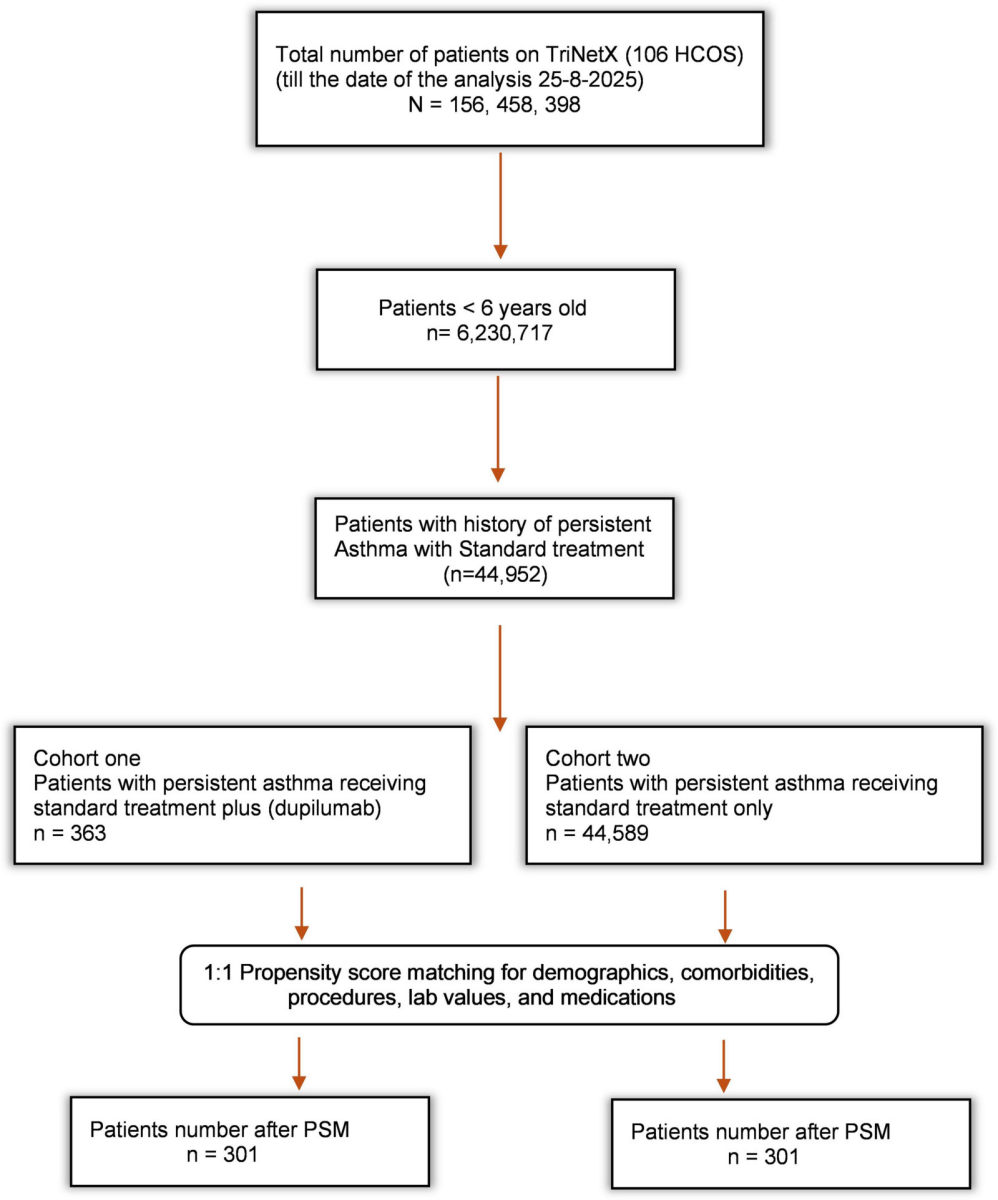
A CONSORT flow diagram for study population selection.

A total of 44,952 patients met the inclusion and exclusion criteria. Of these, 363 patients were assigned to Cohort one and 44,589 to Cohort two. After PSM, each cohort included 301 patients for the final analysis. The study population selection process is shown in Figure 1.

### 3.2 Patient baseline characteristics

Before PSM, children in the DUP cohort were older (3.5 ± 1.3 vs. 2.2 ± 1.4 years) and more likely to be of Black race (37.2% vs. 23.1%). Regarding disease burden, children in the DUP group had higher rates of severe asthma (27.3% vs. 3.3%), more frequent recorded acute exacerbations (32.0% vs. 13.6%), greater OCS use (68.9% vs. 46.1%), and a higher proportion in the eosinophil high-risk category (≥300 cells/µL; 39.9% vs. 10.8%).

After PSM, baseline characteristics were well-balanced between DUP and ST-ONLY groups. Age (3.4 ± 1.3 vs. 3.4 ± 1.3 years), sex distribution (male: 68.8% vs. 67.1%), and race (Black: 35.5% vs. 35.2%) were comparable. Disease burden was also similar, with matched rates of severe asthma (15.0% vs. 15.0%), recorded acute exacerbations (24.3% vs. 23.6%), OCS use (63.5% vs. 61.1%), and eosinophil high-risk indicator (≥300 cells/µL; 34.2% vs. 35.2%) (Table 1).

**Table 1:** Baseline features of children ≤6 years with persistent asthma on dupilumab plus standard treatment vs. standard only.

	Before PSM			After PSM			
	Dupilumab plus standard treatment (cohort one) (*n* = 363)	Standard treatment (cohort two) (*n* = 44,589)	*P*-Value	Dupilumab plus standard treatment (cohort one) (*n* = 301)	Standard treatment (cohort two) (*n* = 301)	*P*-Value	SMD
Demographics							
Age	3.5 ± 1.3	2.2 ± 1.4	<0.001	3.4 ± 1.3	3.4 ± 1.3	1	<0.001
White	157 (43.3%)	23,489 (52.7%)	<0.001	137 (45.5%)	140 (46.5%)	0.806	0.02
Male	250 (68.9%)	27,795 (62.3%)	0.01	207 (68.8%)	202 (67.1%)	0.662	0.036
Black or African American	135 (37.2%)	10,321 (23.1%)	<0.001	107 (35.5%)	106 (35.2%)	0.932	0.007
Diagnosis							
Moderate persistent asthma	240 (66.1%)	17,653 (39.6%)	<0.001	180 (59.8%)	174 (57.8%)	0.619	0.041
Mild persistent asthma	231 (63.60%)	28,505 (63.9%)	0.908	191 (63.5%)	184 (61.1%)	0.556	0.048
Severe persistent asthma	99 (27.3%)	1,468 (3.3%)	<0.001	45 (15.0%)	45 (15.0%)	1	<0.001
Moderate persistent asthma with (acute) exacerbation	116 (32.0%)	6,062 (13.6%)	<0.001	73 (24.3%)	71 (23.6%)	0.848	0.016
Moderate persistent asthma with status asthmaticus	48 (13.2%)	1,464 (3.3%)	<0.001	23 (7.6%)	25 (8.3%)	0.763	0.025
Moderate persistent asthma, uncomplicated	199 (54.8%)	11,625 (26.1%)	<0.001	145 (48.2%)	143 (47.5%)	0.87	0.013
Mild persistent asthma, uncomplicated	195 (53.7%)	21,967 (49.3%)	0.091	167 (55.5%)	159 (52.8%)	0.513	0.053
Mild persistent asthma with (acute) exacerbation	76 (20.9%)	6,815 (15.3%)	0.003	57 (18.9%)	55 (18.3%)	0.834	0.017
Mild persistent asthma with status asthmaticus	29 (8.0%)	1,566 (3.5%)	<0.001	15 (5.0%)	15 (5.0%)	1	<0.001
Mild intermittent asthma, uncomplicated	84 (23.1%)	6,320 (14.2%)	<0.001	73 (24.3%)	70 (23.3%)	0.774	0.023
Mild intermittent asthma	144 (39.7%)	10,894 (24.4%)	<0.001	114 (37.9%)	110 (36.5%)	0.736	0.027
Mild intermittent asthma with (acute) exacerbation	83 (22.9%)	5,781 (13.0%)	<0.001	57 (18.9%)	57 (18.9%)	1	<0.001
Mild intermittent asthma with status asthmaticus	18 (5.0%)	1,048 (2.4%)	0.001	11 (3.7%)	10 (3.3%)	0.824	0.018
Severe persistent asthma, uncomplicated	89 (24.5%)	695 (1.6%)	<0.001	37 (12.3%)	33 (11.0%)	0.611	0.041
Severe persistent asthma with (acute) exacerbation	51 (14.0%)	561 (1.3%)	<0.001	17 (5.6%)	21 (7.0%)	0.503	0.055
Severe persistent asthma with status asthmaticus	30 (8.3%)	346 (0.8%)	<0.001	10 (3.3%)	10 (3.3%)	1	<0.001
Atopic dermatitis	285 (78.5%)	8,347 (18.7%)	<0.001	230 (76.4%)	244 (81.1%)	0.163	0.114
Vasomotor and allergic rhinitis	217 (59.8%)	11,834 (26.5%)	<0.001	165 (54.8%)	166 (55.1%)	0.935	0.007
Medications							
betamethasone	29 (8.0%)	607 (1.4%)	<0.001	22 (7.3%)	20 (6.6%)	0.749	0.026
budesonide	217 (59.8%)	15,199 (34.1%)	<0.001	162 (53.8%)	156 (51.8%)	0.624	0.04
formoterol	148 (40.8%)	4,112 (9.2%)	<0.001	96 (31.9%)	102 (33.9%)	0.603	0.042
salmeterol	52 (14.3%)	1,658 (3.7%)	<0.001	38 (12.6%)	33 (11.0%)	0.528	0.052
beclomethasone	10 (2.8%)	877 (2.0%)	0.282	10 (3.3%)	10 (3.3%)	1	<0.001
ciclesonide	10 (2.8%)	187 (0.4%)	<0.001	10 (3.3%)	10 (3.3%)	1	<0.001
fluticasone	334 (92.0%)	34,694 (77.8%)	<0.001	274 (91.0%)	265 (88.0%)	0.231	0.098
prednisolone	250 (68.9%)	20,556 (46.1%)	<0.001	191 (63.5%)	184 (61.1%)	0.556	0.048
prednisone	24 (6.6%)	1,038 (2.3%)	<0.001	16 (5.3%)	17 (5.6%)	0.858	0.015
The most recent Lab findings							
Eosinophils/100 leukocytes in Blood	6.4 ± 7.7	2.6 ± 4.9	<0.001	6.3 ± 7.0	4.1 ± 4.0	0.001	0.391
Basophils/100 leukocytes in Blood	0.6 ± 0.4	0.4 ± 0.4	<0.001	0.5 ± 0.4	0.5 ± 0.3	0.106	0.198
Leukocytes in Blood	9.8 ± 4.1	12.5 ± 79.5	0.638	9.7 ± 3.6	10.9 ± 5.0	0.025	0.262
Leukocytes in Blood by Automated Count	9.8 ± 4.1	11.4 ± 43.2	0.621	9.9 ± 3.8	11.0 ± 5.0	0.045	0.244
IgE [Units/volume] in Serum or Plasma	938.5 ± 1,637.3	250.1 ± 612.2	<0.001	1,054.0 ± 1,823.6	449.8 ± 914.2	0.014	0.419
Eosinophils <150 cells (low risk)	103 (28.4%)	6,918 (15.5%)	0.315	75 (24.9%)	59 (19.6%)	0.128	0.313
Eosinophils ≥150 and <300cells (intermediate risk)	66 (18.2%)	4,030 (9.0%)	0.269	46 (15.3%)	44 (14.6%)	0.819	0.019
Eosinophils ≥ 300 (high risk)	145 (39.9%)	4,819 (10.8%)	0.711	103 (34.2%)	106 (35.2%)	0.797	0.021

Numerical variables are reported as frequencies (n) and percentages (%).

Continuous variables are reported as mean ± SD.

PSM, propensity score matching; SMD, standardized mean difference; SD, standard deviation.

### 3.3 Outcomes

**Figure 2:**
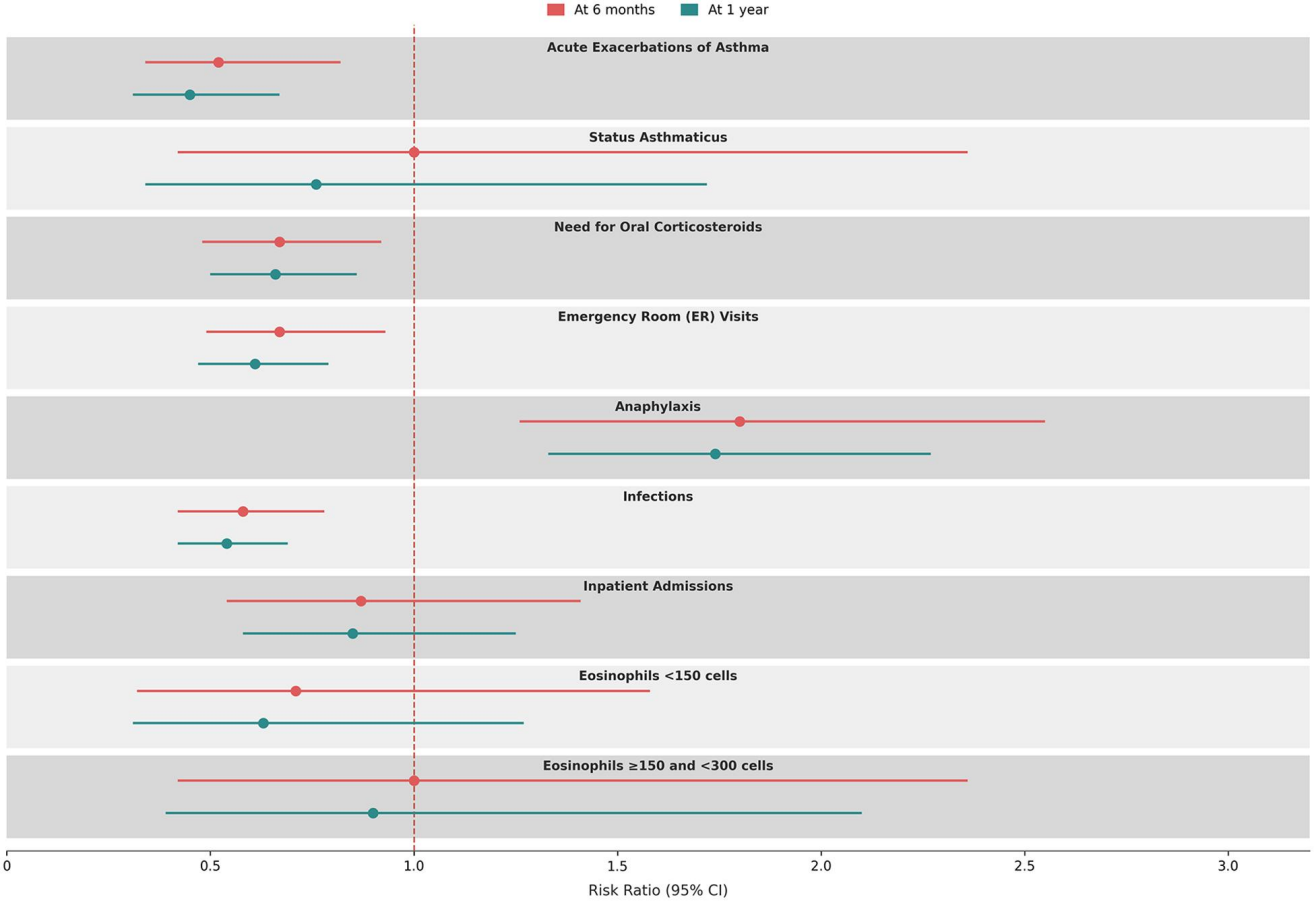
A forest plot comparing dupilumab therapy alongside standard treatment vs. standard treatment alone in children ≤6 years with persistent asthma at 6 and 12 months.

Compared to the ST-ONLY group, the DUP group was significantly associated with reduced risk of acute exacerbations at 6 months (RR 0.53, 95% CI: 0.34–0.82, *p* = 0.004) and at 1 year (RR 0.46, 95% CI: 0.32–0.65, *p* < 0.001). The DUP group was associated with a reduced need for OCS at 6 months (RR 0.67, 95% CI: 0.48–0.92, *p* = 0.015) and at 1 year (RR 0.66, 95% CI: 0.50–0.86, *p* = 0.002). ER visits and infections similarly showed significant reductions in the DUP group across all time points (all *p* < 0.05). Conversely, the risk of anaphylaxis was higher in the DUP group at 6 months (RR 1.80, 95% CI: 1.27–2.56, *p* = 0.001) and at 1 year (RR 1.74, 95% CI: 1.32–2.28, *p* < 0.001). No significant differences were observed for status asthmaticus, eosinophil-based risk indicators, or inpatient admissions. Patient counts ranged from 1 to 9, are reported as 10 in TriNetX to protect privacy. As a result, any results with counts of 10, even if significant (such as the eosinophil high-risk category), are unreliable (Table 2, Figure 2).

**Table 2:** Outcomes of children aged ≤6 years with persistent asthma: dupilumab plus standard treatment vs. standard treatment alone.

Outcomes	Time point	Study cohorts	Risk analysis				
		Overall (*N*= 602)	Dupilumab plus standard treatment (Cohort One) (*n* = 301)	Standard treatment (Cohort two) (*n* = 301)	Risk difference	Risk Ratio (95% confidence interval)	*P*-value
Primary Outcomes							
Acute Exacerbations of Asthma	6 months	**78 (13.0%)**	**27 (9.0%)**	**51 (16.9%)**	**−0.08**	**0.52 (0.34, 0.82)**	**0.004**
	12 months	**105 (17.4%)**	**33 (11.0%)**	**72 (23.9%)**	**−0.13**	**0.45 (0.31–0.67)**	**<0.001**
Status Asthmaticus	6 months	20 (3.3%)	10 (3.3%)	10 (3.3%)	0	1 (0.42, 2.36)	1
	12 months	23 (3.8%)	10 (3.3%)	13 (4.3%)	−0.01	0.76 (0.34, 1.72)	0.524
Need for Oral Corticosteroids	6 months	**122 (20.3%)**	**49 (16.3%)**	**73 (24.3%)**	**−0.08**	**0.67 (0.48, 0.92)**	**0.015**
	12 months	**166 (27.6%)**	**66 (21.9%)**	**100 (33.2%)**	**−0.113**	**0.66 (0.50, 0.86)**	**0.002**
Secondary Outcomes							
Emergency room (ER) visit	6 months	**124 (20.6%)**	**50 (16.6%)**	**74 (24.6%)**	**−0.08**	**0.67 (0.49, 0.93)**	**0.016**
	12 months	**179 (29.7%)**	**68 (22.6%)**	**111 (36.9%)**	**−0.143**	**0.61 (0.47, 0.79)**	**<0.001**
Anaphylaxis	6 months	**112 (18.6%)**	**72 (23.9%)**	**40 (13.3%)**	**0.106**	**1.80 (1.26, 2.55)**	**0.001**
	12 months	**170 (28.2%)**	**108 (35.9%)**	**62 (20.6%)**	**0.153**	**1.74 (1.33, 2.27)**	**<0.001**
Infections	6 months	**139 (23.1%)**	**51 (16.9%)**	**88 (29.2%)**	**−0.123**	**0.58 (0.42, 0.78)**	**<0.001**
	12 months	**198 (32.9%)**	**70 (23.3%)**	**128 (42.5%)**	**−0.193**	**0.54 (0.42, 0.69)**	**<0.001**
Inpatient Admissions	6 months	62 (10.3%)	29 (9.6%)	33 (11.0%)	−0.013	0.87 (0.54, 1.41)	0.592
	12 months	91 (15.1%)	42 (14.0%)	49 (16.3%)	−0.023	0.85 (0.58–1.25)	0.426
Eosinophils <150 cells (low risk)	6 months	24 (4.0%)	10 (3.3%)	14 (4.7%)	−0.013	0.71 (0.32, 1.58)	0.405
	12 months	31 (5.1%)	12 (4.0%)	19 (6.3%)	−0.023	0.63 (0.31, 1.27)	0.197
Eosinophils ≥150 and <300cells (intermediate risk)	6 months	20 (3.3%)	10 (3.3%)	10 (3.3%)	0	1 (0.42, 2.36)	1
	12 months	21 (3.5%)	10 (3.3%)	11 (3.7%)	−0.003	0.90 (0.39, 2.10)	0.824
Eosinophils ≥ 300 (high risk)	6 months	10 (1.7%)	10 (3.3%)	0	0.033	_	0.001
	12 months	10 (1.7%)	10 (3.3%)	0	0.033	_	0.001

Numerical variables are reported as frequencies (n) and percentages (%).

Outcomes with values of 1 to 10 patients are rounded to 10 to protect patients’ confidentiality.

Bold font highlights significant outcomes.

## 4 Discussion

This study is the first to assess dupilumab in children ≤6 years, a population excluded from prior RCTs. The first follow-up period was set at 6 months to allow sufficient time for the therapeutic effect to emerge. The cohort comparability was ensured among 44,952 children by adjusting for baseline asthma severity, its complications, allergic comorbidities, and prior OCS use.

Children in the DUP group showed consistently better asthma control compared with the standard treatment alone group (ST-ONLY). The therapy led to fewer exacerbations, less reliance on OCS, and a broadly reduced acute healthcare burden, all indicating improved disease management and reduced burden of asthma. Moreover, fewer ER visits and infections further support the clinical benefit of dupilumab in this population. However, the DUP group was associated with a higher risk of anaphylaxis, which represents a significant safety concern that needs careful monitoring. On the other hand, no apparent differences were noted in terms of status asthmaticus or inpatient admissions.

In children aged ≤6 years, dupilumab therapy was associated with a 51% reduction in the relative risk of acute exacerbations at 6 months and a 59% reduction at 1 year. These findings are consistent with the VOYAGE trial in children aged 6–11 years, as well as with pivotal phase 3 trials including QUEST, VENTURE, and their long-term extensions (10, 13, 21–23). Notably, our study extends these benefits to real-world evidence in the pediatric population, where disease phenotypes and comorbidities are more heterogeneous than in randomized controlled trials. OCS prescription rates were significantly reduced at both follow-up intervals (RR 0.67 and 0.66; *p* < 0.05), reinforcing the role of dupilumab in mitigating long-term steroid dependence in this vulnerable population. This steroid-sparing effect is of high clinical relevance, as systemic OCS exposure in early childhood is associated with substantial adverse consequences, including growth impairment and immunosuppression (24–26). ER visits were markedly lower in the dupilumab group at 6 months, accompanied by a reduction in the composite infection endpoint — which included acute upper respiratory infections alongside unspecified-site bacterial and viral infections — suggesting a broader attenuation of infectious morbidity, though a purely respiratory attribution cannot be made (1, 27). It should be acknowledged that both cohorts received background standard-of-care therapy throughout the observation period; the clinical improvements observed in the DUP group therefore represent the incremental benefit conferred by dupilumab above this baseline. No pharmacokinetic interactions between dupilumab and ICS, LABA, or LTRA have been identified to date; however, a purely synergistic effect — whereby dupilumab-mediated suppression of type 2 inflammation amplifies the local airway effects of ICS — cannot be excluded in this observational setting.

Regarding safety, the dupilumab cohort demonstrated a significantly higher incidence of anaphylaxis at both time points; however, causal attribution requires careful contextualisation. The retrospective design precludes establishing a temporal relationship between dupilumab administration and individual anaphylaxis events, as recorded episodes may precede or be entirely unrelated to drug exposure. Moreover, food allergy — a well-recognised anaphylaxis trigger in this age group and a central component of the atopic march — was not included as a matching covariate, representing a potential source of residual confounding. Ascertainment bias may further contribute, as children receiving dupilumab likely undergo more frequent clinical monitoring, increasing the probability of detecting and coding allergic events. Pharmacovigilance data from the FDA Adverse Event Reporting System suggest a lower anaphylaxis signal for dupilumab compared with omalizumab, raising the possibility that some excess diagnoses in our cohort reflect coding artefacts rather than a direct drug effect (28–30). ICD-10 coding cannot differentiate drug-induced hypersensitivity from IgE-mediated reactions to food or environmental triggers (30, 31) — a distinction of particular relevance in a cohort where atopic multimorbidity was the norm rather than the exception. These findings underscore the need for structured post-injection observation protocols analogous to those established for omalizumab, with emergency management resources available at the point of administration. Prospective studies and pharmacovigilance registries remain essential to accurately characterise the true anaphylaxis risk in this age group (28, 30). Across all three eosinophil risk categories, no statistically significant between-group differences were observed at either timepoint, with the exception of a nominally significant finding in the high-risk category (≥300 cells/µL) at both 6 and 12 months; as these counts fell below the TriNetX privacy threshold and were reported as rounded values, no conclusions regarding eosinophilia trajectory can be drawn from these data (32). No significant differences were observed in status asthmaticus, inpatient admissions, or IgE levels. However, absolute numbers for severe outcomes were consistently lower in the dupilumab group, suggesting a non-significant protective trend. Overall, dupilumab therapy was associated with substantial improvements in asthma control in children ≤6 years, including reduced exacerbation frequency, OCS use, ER visits, and overall infection burden, though no statistically significant reduction in inpatient admissions was demonstrated. The eosinophilia findings and elevated anaphylaxis risk appear manageable within the known safety framework, but warrant ongoing clinical vigilance (33). Limited follow-up may have constrained the detection of rare severe events such as status asthmaticus.

## 5 Strengths and limitations

### 5.1 Strengths

This study's primary strength is its use of a large, multi-institutional real-world database, allowing for a broad and diverse population of children < 6 years with persistent asthma, a group often excluded from previous RCTs. Propensity score matching minimized baseline imbalances, reducing confounding risk. As one of the first studies to provide comparative data on dupilumab in this very young age group, it directly addresses a significant evidence gap.

### 5.2 Limitations

The retrospective design introduces potential for residual confounding despite propensity score matching. Although racial distribution was well-balanced after PSM (Black: 35.5% vs. 35.2%, SMD 0.007), race as a demographic variable does not fully capture the structural determinants it reflects — including disparities in healthcare access, environmental allergen burden, and socioeconomic circumstance — all of which are known to influence asthma outcomes independently. Residual confounding through these unmeasured pathways therefore cannot be excluded, and the relatively high proportion of Black children in both cohorts should be considered when assessing the generalizability of these findings. Reliance on ICD-10 codes and electronic medical records may lead to underreporting or misclassification of outcomes. Additionally, asthma diagnosis in children under six years was ascertained solely from ICD-10-CM codes and cannot be verified against clinical criteria or spirometry. Given the well-recognized diagnostic uncertainty in this age group — where recurrent wheezing is common and does not always signify true persistent asthma — some degree of diagnostic misclassification may have occurred. Key variables like treatment adherence, dosing, and environmental exposures were unavailable, limiting adjustment for influential factors. Medication data in TriNetX are derived from prescription records in the EHR and do not confirm dispensing or adherence. It is therefore possible that some patients classified as receiving standard therapy may not have been consistently taking their prescribed medications. The infection outcome represented a composite of acute upper respiratory infections and unspecified-site bacterial and viral infection codes; as such, it cannot be characterised as exclusively respiratory. The contribution of non-respiratory infections to the observed between-group difference cannot be quantified, which limits the specificity of interpretation. Observed anaphylaxis differences require cautious interpretation due to potential coding errors or reporting bias. Emergency room visits and inpatient admissions were captured as all-cause encounter events within TriNetX and could not be restricted to respiratory or asthma-related diagnoses. The observed reduction in ER visits in the dupilumab cohort should therefore not be interpreted as reflecting exclusively asthma-driven healthcare utilisation. Nevertheless, as propensity score matching balanced both cohorts across a broad range of diagnoses and comorbidities, the comparison represents two groups with similar overall medical burden, and the between-group difference in acute healthcare encounters remains a meaningful, if non-specific, indicator of clinical benefit. Food allergy was not captured as a covariate in the propensity score matching model. Given its prevalence in the atopic march at these ages, and the fact that anaphylaxis was one of the study endpoints, the possibility that some anaphylaxis events were attributable to IgE-mediated food-triggered reactions rather than dupilumab exposure cannot be excluded (31). Moreover, Eosinophilic esophagitis (EoE) was not included as a specific diagnostic covariate in the propensity score matching model, and its prevalence was not systematically extracted from the TriNetX database for either cohort. This is a recognized limitation, particularly given published evidence that EoE was identified in 23.4% of children with severe or poorly controlled asthma (34). Consequently, the distribution of EoE across the two matched cohorts cannot be compared or reported. The inability to stratify by prescribing indication or to account for EoE prevalence represents a potential source of residual confounding and indication bias that propensity score matching could not fully resolve. Future studies using TriNetX or comparable databases should explicitly extract EoE as a diagnostic and matching covariate when evaluating dupilumab in young children. Furthermore, TriNetX does not record the season of clinical assessment, nor does it distinguish perennial from seasonal allergic rhinitis. Temporal variation in allergen exposure between the study cohorts may therefore represent an additional source of bias that was not accounted for in this analysis. The one-year follow-up may not capture long-term outcomes or rare adverse events. Furthermore, as TriNetX does not maintain a structured individual-level follow-up record, treatment discontinuation or dropout from the dupilumab cohort during the observation window cannot be verified or quantified, which may affect the accuracy of the exposure classification over time. Additionally, LABA prescriptions identified in this dataset could not be linked to the prescribing clinician's specialty, the patient's exact age at the time of prescribing, or the clinical context in which they were issued. Current guidelines recommend restricting LABA use in children under 6 years to exceptional circumstances under specialist supervision, with most regulatory approvals applying to those aged 4 years and older. Whether the LABA prescriptions captured in this cohort complied with these age-specific restrictions cannot be determined from the TriNetX dataset, representing a limitation that may affect the characterisation of standard therapy in our study population. Dupilumab prescriptions in the DUP cohort could not be linked to a specific clinical indication at the encounter level. Although dupilumab prescriptions in this cohort could not be linked to a specific clinical indication at the encounter level, both matched cohorts carried a similar burden of atopic dermatitis (DUP: 76.4% vs. ST-ONLY: 81.1%; SMD 0.114), rendering differential atopic skin disease management an unlikely explanation for the between-group differences in ER visits and OCS use. The observed improvements are therefore more plausibly attributable to dupilumab's pharmacological suppression of shared type 2 inflammatory pathways — spanning both atopic dermatitis and asthma — rather than to an unbalanced confound from comorbid skin disease, though a precise attribution between indications cannot be made from observational data (18, 19). Finally, differences from randomized controlled trials (e.g., double-masked, placebo-controlled) affect comparability and generalizability, and selection bias in real-world cohorts cannot be entirely excluded.

## 6 Clinical implications

These findings support early dupilumab introduction as concurrent therapy for children in this age group with persistent, uncontrolled asthma and a type 2 inflammatory phenotype. This could reduce systemic corticosteroid reliance and mitigate its long-term sequelae. The data may inform future guideline updates (e.g., AAP, GINA) for younger children. However, generalizability to broader populations and long-term safety in very young children require further study. Regarding long-term safety, clinicians should exercise caution when initiating dupilumab in children ≤6 years, given the absence of dedicated trial data in this age group and the inherent constraints of a 12-month observational window. Eosinophil counts across all risk categories did not differ significantly between cohorts at either timepoint, consistent with dupilumab's established safety profile (32). The elevated anaphylaxis signal, however, warrants particular consideration: our study cannot determine whether these events were causally related to dupilumab, attributable to co-existing food allergy or other atopic triggers, or incidental to the observation period. Clinicians should prospectively document and phenotype any anaphylaxis events in relation to dupilumab administration, with particular vigilance in children carrying a high atopic burden or known food allergy. Adrenaline availability at the point of care and structured post-injection observation are advisable precautions. Longer-term prospective registries are urgently needed to clarify the true anaphylaxis risk and overall safety durability in this age group. Ongoing surveillance and prospective studies are needed to confirm benefits and optimize patient selection.

## 7 Conclusion

This real-world analysis shows dupilumab therapy alongside standard treatment for children ≤6 years with persistent asthma significantly reduces exacerbations, need for OCS, and ER visits. An elevated anaphylaxis risk was observed in the dupilumab cohort, warranting structured clinical monitoring. Other outcomes were comparable. These findings support dupilumab's clinical benefit, but observational data limitations require cautious interpretation and ongoing monitoring.

## Ethics consideration

This retrospective study is exempt from informed consent, as it constitutes a secondary analysis of existing de-identified data and does not involve intervention or interaction with human subjects. Patient data are de-identified in accordance with the standard defined in Section §164.514(a) of the HIPAA Privacy Rule. The de-identification process is attested to through a formal determination by a qualified expert as defined in Section §164.514(b)(1) of the HIPAA Privacy Rule; this formal determination was last refreshed in December 2020. Participating healthcare organizations maintain full compliance with these standards. The study adhered to the Strengthening the Reporting of Observational Studies in Epidemiology (STROBE) guidelines and the Publication Guidelines specified by the TriNetX (16, 17).

## Data Availability

Publicly available datasets were analyzed in this study. This data can be found here: https://trinetx.com.
