# Supplementary material for: Real-world effectiveness and safety of dupilumab therapy in children ≤6 years with uncontrolled persistent asthma: a propensity-matched retrospective cohort study
Source: Front Pediatr. 2026 Jun 15;14:1808733. doi: 10.3389/fped.2026.1808733 (PMC13311024; doi:10.3389/fped.2026.1808733)
Supplement: Supplementary file 1 [file Supplementaryfile1.docx]

**Supplementary Materials**

**Dupilumab Add-On Therapy in Children ≤6 years with Uncontrolled Asthma on Standard Therapy: A Real-world Retrospective Analysis**

**Table legends**

**Table S1**: Participants’ eligibility with the relevant codes

**Table S2**: Baseline selected variables with their relevant codes

**Table S3**: Outcome definition and codes used for identification

**Figure legends**

**Figure S1**: Density curves depicting the process of propensity score matching

| **Supplementary Table 1:** Participants’ eligibility with the relevant codes | | | | | | | | | |  |
| --- | --- | --- | --- | --- | --- | --- | --- | --- | --- | --- |
| **Dupilumab Plus Standard Treatment (cohort one)** | | | | | **Standard Treatment (cohort two)** | | | | |  |
| **Ungrouped terms** | | | | | **Ungrouped terms** | | | | |  |
| Must have | | demographics | Age | Age (between 0 and 6 years (most recent occurrence)) | Must have | | demographics | Age | Age (between 0 and 6 years (most recent occurrence)) |  |
| **Group 1** | | | | | **Group 1** | | | | |  |
| • date constraint: This group occurred before 1 year ago | | | | | • date constraint: The terms in this group occurred at any time | | | | |  |
| • event relationship: Any instance of biological treatment occurred within 1 year on or after any instance of persistent asthma | | | | | • event relationship: Any instance of no biological treatment occurred within 6 months before or up to 1 year after any instance of persistent asthma | | | | |  |
| Group 1A | must have any of | Diagnosis | UMLS:ICD10CM:J45.3 UMLS:ICD10CM:J45.4 UMLS:ICD10CM:J45.5 | Mild persistent asthma Moderate persistent asthma Severe persistent asthma | Group 1A | must have any of | Diagnosis | UMLS:ICD10CM:J45.3 UMLS:ICD10CM:J45.4 UMLS:ICD10CM:J45.5 | Mild persistent asthma Moderate persistent asthma Severe persistent asthma |  |
| Group 1B | must have | Medications | NLM:RXNORM:1876376 | dupilumab | Group 1B | cannot have any of | Medications | NLM:RXNORM:2587789 NLM:RXNORM:1720597 NLM:RXNORM:302379 NLM:RXNORM:1989100 NLM:RXNORM:1876376 | tezepelumab mepolizumab omalizumab benralizumab dupilumab |  |
|  |  |  |  |  |  |  |  |  |  |  |
|  |  |  |  |  |  |  |  |  |  |  |
| **Group 2** | | | | | **Group 2** | | | | |  |
| • date constraint: This group occurred before 1 year ago | | | | | • date constraint: This group occurred before 1 year ago | | | | |  |
| •event relationship: Any instance of standard therapy occurred within 1 year on or after any instance of persistent asthma | | | | | •event relationship: Any instance of standard therapy occurred within 1 year on or after any instance of persistent asthma | | | | |  |
| Group 2A | must have | Diagnosis | UMLS:ICD10CM:J45.3 UMLS:ICD10CM:J45.4 UMLS:ICD10CM:J45.5 | Mild persistent asthma Moderate persistent asthma Severe persistent asthma | Group 2A | must have | Diagnosis | UMLS:ICD10CM:J45.3 UMLS:ICD10CM:J45.4 UMLS:ICD10CM:J45.5 | Mild persistent asthma Moderate persistent asthma Severe persistent asthma |  |
|  |  |  |  |  |  |  |  |  |  |  |
| Group 2B | must have | Medications | NLM:RXNORM:25255 NLM:RXNORM:36117 NLM:RXNORM:1347 NLM:RXNORM:19831 NLM:RXNORM:274964 NLM:RXNORM:41126 NLM:RXNORM:121047 NLM:RXNORM:1806903 NLM:RXNORM:88249 | formoterol salmeterol beclomethasone budesonide ciclesonide fluticasone propionate propylene glycol dipropionate montelukast | Group 2B | must have | Medications | NLM:RXNORM:25255 NLM:RXNORM:36117 NLM:RXNORM:1347 NLM:RXNORM:19831 NLM:RXNORM:274964 NLM:RXNORM:41126 NLM:RXNORM:121047 NLM:RXNORM:1806903 NLM:RXNORM:88249 | Formoterol Salmeterol beclomethasone Budesonide Ciclesonide Fluticasone Propionate propylene glycol dipropionate montelukast |  |
|  |  |  |  |  |  |  |  |  |  |  |

| **Supplementary Table 2:** Baseline selected variables with their relevant codes | | |
| --- | --- | --- |
| **Category** | **Code** | **Code Description** |
| **Demographics** | 2106-3 | White |
|  | 2076-8 | Native Hawaiian or Other Pacific Islander |
|  | 1002-5 | American Indian or Alaska Native |
|  | 2054-5 | Black or African American |
|  | 2028-9 | Asian |
| **Diagnosis** | J45.4 | Moderate persistent asthma |
|  | J45.3 | Mild persistent asthma |
|  | J45.5 | Severe persistent asthma |
|  | J45.41 | Moderate persistent asthma with (acute) exacerbation |
|  | J45.42 | Moderate persistent asthma with status asthmaticus |
|  | J45.40 | Moderate persistent asthma, uncomplicated |
|  | J45.30 | Mild persistent asthma, uncomplicated |
|  | J45.31 | Mild persistent asthma with (acute) exacerbation |
|  | J45.32 | Mild persistent asthma with status asthmaticus |
|  | J45.20 | Mild intermittent asthma, uncomplicated |
|  | J45.2 | Mild intermittent asthma |
|  | J45.21 | Mild intermittent asthma with (acute) exacerbation |
|  | J45.22 | Mild intermittent asthma with status asthmaticus |
|  | J45.50 | Severe persistent asthma, uncomplicated |
|  | J45.51 | Severe persistent asthma with (acute) exacerbation |
|  | J45.52 | Severe persistent asthma with status asthmaticus |
|  | L20 | Atopic dermatitis |
|  | J30 | Vasomotor and allergic rhinitis |
| **Medication** | 1514 | betamethasone |
|  | 19831 | Budesonide |
|  | 25255 | Formoterol |
|  | 36117 | salmeterol |
|  | 1347 | beclomethasone |
|  | 274964 | ciclesonide |
|  | 41126 | fluticasone |
|  | 8638 | prednisolone |
|  | 8640 | prednisone |
| **Laboratory** | 9007 | Eosinophils/100 leukocytes in Blood |
|  | 9006 | Basophils/100 leukocytes in Blood |
|  | 9015 | Leukocytes [volume] in Blood |
|  | 6690-2 | Leukocytes [volume] in Blood by Automated count |
|  | 26464-8 | Leukocytes [volume] in Blood |
|  | 19113-0 | IgE [Units/volume] in Serum or Plasma |
|  | LG5901-6 | IgE [Units/volume] in Serum, Plasma, or Blood |
|  | LG32849-8 | Eosinophils [volume] in Blood |

| **Supplementary Table 3:** Outcome definition and codes used for identification | | | |
| --- | --- | --- | --- |
| **Outcome** | **Coding system** | **Code** | **Code description** |
| **Primary Outcomes** | | | |
| **Acute Exacerbations of Asthma** | ICD10CM | J45.41 | Moderate persistent asthma with (acute) exacerbation |
|  | ICD10CM | J45.31 | Mild persistent asthma with (acute) exacerbation |
|  | ICD10CM | J45.51 | Severe persistent asthma with (acute) exacerbation |
| **status Asthmaticus** | ICD10CM | J45.42 | Moderate persistent asthma with status asthmaticus |
|  | ICD10CM | J45.32 | Mild persistent asthma with status asthmaticus |
|  | ICD10CM | J45.52 | Severe persistent asthma with status asthmaticus |
| **Need for oral corticosteroids** | RXNORM | 8638 | Prednisolone |
|  | RXNORM | 8640 | Prednisone |
| **Secondary Outcomes** | | | |
| **Emergency Hospitalizations** | CPT | 1013711 | Emergency Department Services |
|  | CPT | 99281 | Emergency department visit for the evaluation and management of a patient that may not require the presence of a physician or other qualified health care professional |
|  | CPT | 99282 | Emergency department visit for the evaluation and management of a patient, which requires a medically appropriate history and/or examination and straightforward medical decision making |
|  | CPT | 99283 | Emergency department visit for the evaluation and management of a patient, which requires a medically appropriate history and/or examination and low level of medical decision making |
|  | CPT | 99284 | Emergency department visit for the evaluation and management of a patient, which requires a medically appropriate history and/or examination and moderate level of medical decision making |
|  | CPT | 99285 | Emergency department visit for the evaluation and management of a patient, which requires a medically appropriate history and/or examination and high level of medical decision making |
|  | HL7V3 | VisitType:EMER | Visit: Emergency |
| **Eosinophils <150 cells** | TNX | LG32849-8 | Eosinophils [volume] in Blood (between 0.00 and 0.15 10*3/uL (most recent occurrence)) |
| **Eosinophils >=150 and <300cells** | TNX | LG32849-8 | Eosinophils [volume] in Blood (between 0.15 and 0.30 10*3/uL (most recent occurrence)) |
| **Eosinophils >= 300** | TNX | LG32849-8 | Eosinophils [volume] in Blood (at least 300.00 10*3/uL (most recent occurrence)) |
| **Anaphylaxis** | ICD10CM | T78.2 | Anaphylactic shock, unspecified |
|  | ICD10CM | T88.6 | Anaphylactic reaction due to adverse effect of correct drug or medicament properly administered |
|  | RXNORM | 3992 | Epinephrine |
| **infections** | ICD10CM | A49 | Bacterial infection of unspecified site |
|  | ICD10CM | B34 | Viral infection of unspecified site |
|  | ICD10CM | J00-J06 | Acute upper respiratory infections |
| **Inpatient admissions** | HL7V3.0 | VisitType:ACUTE | Visit: Inpatient Acute |
|  | HL7V3.0 | VisitType:IMP | Visit: Inpatient Encounter |
|  | HL7V3.0 | VisitType:NONAC | Visit: Inpatient Non-acute |
|  | HL7V3.0 | VisitType:SS | Visit: Short Stay |


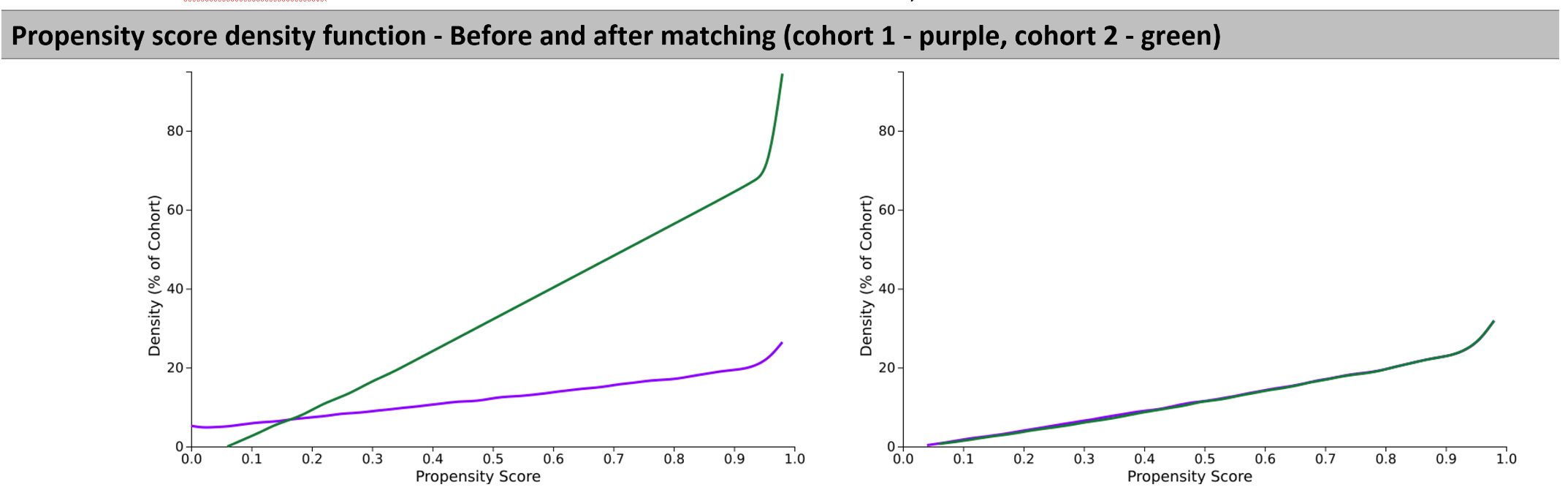


Figure S1: Density curves depicting the process of propensity score matching
